# Associations and Dissociations between Oculomotor Readiness and Covert Attention

**DOI:** 10.3390/vision3020017

**Published:** 2019-05-07

**Authors:** Soazig Casteau, Daniel T. Smith

**Affiliations:** Department of Psychology, Durham University, Durham DH1 3HP, UK

**Keywords:** attention, covert, oculomotor readiness hypothesis, premotor theory, exogenous, endogenous, eye abduction

## Abstract

The idea that covert mental processes such as spatial attention are fundamentally dependent on systems that control overt movements of the eyes has had a profound influence on theoretical models of spatial attention. However, theories such as Klein’s Oculomotor Readiness Hypothesis (OMRH) and Rizzolatti’s Premotor Theory have not gone unchallenged. We previously argued that although OMRH/Premotor theory is inadequate to explain pre-saccadic attention and endogenous covert orienting, it may still be tenable as a theory of exogenous covert orienting. In this article we briefly reiterate the key lines of argument for and against OMRH/Premotor theory, then evaluate the Oculomotor Readiness account of Exogenous Orienting (OREO) with respect to more recent empirical data. These studies broadly confirm the importance of oculomotor preparation for covert, exogenous attention. We explain this relationship in terms of reciprocal links between parietal ‘priority maps’ and the midbrain oculomotor centres that translate priority-related activation into potential saccade endpoints. We conclude that the OMRH/Premotor theory hypothesis is false for covert, endogenous orienting but remains tenable as an explanation for covert, exogenous orienting.

## 1. Introduction

Covert spatial attention allows us to select important and/or behaviourally relevant visual inputs by enhancing signals arising from attended locations and suppressing signals from unattended locations [1] without actually moving the eyes to that location. Despite many advances in understanding the cognitive processes involved in spatial attentional selection, an enduring issue is the mechanism by which attention is moved from one location to another. It is generally agreed that the orienting of spatial attention can occur in an automatic ‘exogenous’ mode in response to salient external events (e.g., the flashing lights of an emergency services vehicle) or a controlled ‘endogenous’ mode in response to the observer’s goals (e.g., systematically scanning the road ahead to check for hazards) [2], and that these systems are partially dissociable [3]. It is also widely accepted that eye movements (‘overt’ shifts of attention) are preceded by a covert shift of attention to the saccade goal, known as ‘pre-saccadic attention’. However, there is a long-running debate concerning the relationship between the mental process involved in covert orienting of attention (i.e., attending to things that are not being gazed at), and those involved in overt orienting of attention (i.e., orienting the eye to the stimulus of interest) [4]. One proposal, originally known as the Oculomotor Readiness Hypothesis (OMRH) [5] and later as Premotor Theory (PMT) [6], proposed a complete functional overlap between spatial attention and oculomotor control. OMRH/PMT is often used as shorthand to refer to the general idea that covert attention is, in some way, linked to the oculomotor system. However, this usage does not do full justice to the OMRH/PMT theory, which makes clear and testable predictions about the precise relationship between oculomotor control and covert spatial attention. More specifically, OMRH/PMT holds that the programming of a saccade is both necessary and sufficient for covert orienting of attention [7].

Despite being the original proponents of OMRH, Klein and colleagues concluded that endogenous attention was in fact independent of saccade programming [5,8], although they speculated that OMRH may still hold for exogenous attention. Subsequently, a number of other proposals suggesting differing degrees of overlap between attention and saccade control have been put forward [9,10,11]. Following the work of Klein and colleagues, we have pursued the idea that the relationship between covert attention and saccade programming may indeed be dependent on the mode of orienting, such that OMRH/PMT was only true when the exogenous mode of orienting was engaged [4]. In this review we outline the main lines of argument for and against OMRH/PMT as a theory of endogenous covert orienting, then explain why we believe that OMRH/PMT is false for endogenous covert orienting, but remains tenable as a theory of exogenous, covert orienting.

## 2. The Case for OMRH/PMT

The case for OMRH/PMT draws on three main lines of evidence. Firstly, there is clear evidence that saccadic eye movements are preceded by a mandatory ‘pre-saccadic’ shift of attention [12,13,14,15,16,17,18] and a more efficient distractor suppression at non-saccade goals [19]. This pre-saccadic attentional facilitation is clearly tied to the programming of an eye movement, as the effect grows larger with proximity with saccade onset [20,21] and occurs even when the participant expects the probe to appear opposite the saccade goal, implying that programming an eye movement is sufficient to trigger a shift of covert attention [13]. Furthermore, shifts of attention appear to affect the trajectory of saccadic eye movements, consistent with the idea that shifts of attention activate a saccade plan [16,22,23].

**Figure 1:**
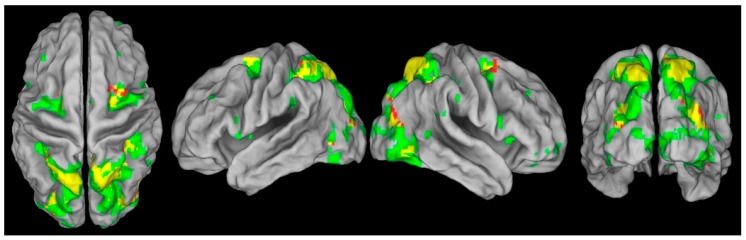
In red are the areas of the brain that are significantly activated in the covert shift of attention task and in green the areas of the brain significantly activated in the overt shift of attention task. In yellow are the areas of the brain activated in both the overt and the covert shift of attention task. Reproduced with permission from [24].

Secondly, eye movements and covert shifts of attention appear to activate similar networks of brain areas, including the Frontal Eye Fields (FEF), the Lateral Intraparietal cortex, and the Superior Colliculi (SC) [24,25,26,27,28,29](see Figure 1), and lesions to these brain areas are associated with deficits of both covert orienting and saccade control [30,31,32,33,34,35,36]. Moreover, electrical stimulation of FEF neurons in non-human primates elicited fixed-vector saccadic eye movements, and subthreshold stimulation of the same neurons significantly enhanced perceptual discrimination, even though the monkey was still centrally fixating [37,38]. Using a similar methodology, Moore and colleagues also demonstrated that stimulation of FEF modulated the sensitivity of neurons in V4, an area of the visual cortex that codes for colour, orientation and spatial frequency, and whose visual receptive fields overlap with the motor field [39,40]. The effect of FEF microstimulation on neural responses in V4 was analogous to that observed when the monkey endogenously attended the location [39]. These data suggest a causal role for saccade programming in covert attention, as predicted by OMRH/PMT.

**Figure 2:**
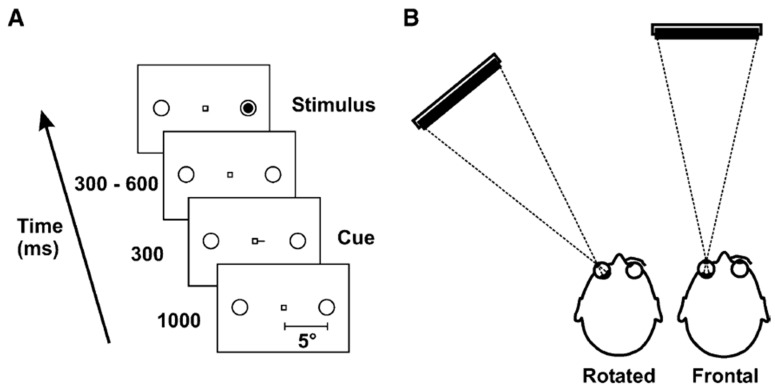
Experimental setup for the eye-abduction paradigm used by Craighero et al. (2004). Reproduced with permission from [42].

A third line of argument draws on studies in which eye movements are impaired, experimentally restricted, or experimentally modulated. For example, Craighero, Carta and Fadiga [41] observed that patients with a palsy of the VI^th^ cranial nerve were unable to covertly orient attention only when viewing stimuli with their palsied eye, suggesting that the endogenous shift of attention was impaired when viewing with the damaged eye but not when viewing with the intact one. In line with this study, Craighero, Nascimben and Fadiga [42] used an eye abduction paradigm (see Figure 2), where saccadic eye movement programming is disrupted by forcing healthy participants to rotate the eye by 40° into the temporal hemifield. In their experiment participants were presented with a classical Posner cueing task in which a central predictive cue (i.e., a bar attached to the fixation square indicating left or right) indicated in 70% of the cases the accurate position of the upcoming target, which could be either in the nasal hemispace (i.e., at a position that can be reached by a saccadic eye movement) or in the temporal hemispace (i.e., outside a position reachable by a saccadic eye movement). Visual acuity remained unaffected but the attentional benefits typically observed with valid cues were reduced when stimuli were presented in the temporal/eye movement restricted hemispace but not when presented in the nasal hemispace. The authors concluded that, consistent with Premotor theory, covert orienting of attention is subject to the limitations of the saccadic system such that attention cannot be deployed at a location that cannot become the goal of a saccadic eye movement. This led to the proposal that covert attention and saccadic eye movements share the same ‘stop limit’, which is the range of eye movements, also referred to as Effective Oculomotor Range (EOMR).

Other studies have used the saccadic adaptation technique to dissociate the perceived position of a saccade target from the actual endpoint of the eye movement. In saccadic adaptation tasks the participant makes a saccade to a peripheral stimulus, but during the saccade the stimulus jumps to a new position (double-step task) [43]. At the start of the experiment the participant initially moves to the original stimulus position then, unconsciously, makes corrective eye movements towards the second stimulus position. However, over the course of many trials they adapt the amplitude of the saccade to ensure it lands at the final position of the stimulus rather than its original position (for a review, see [44]). OMRH/PMT predicts that saccadic adaptation should also result in the adaptation of covert shifts of attention, such that the locus of attention should be at the final stimulus position, not the starting position. To test whether attention focus is shifted towards the saccade target or the final eye position, Ditterich et al. [45] asked participants to make a saccade towards a peripheral location and, before the first saccade onset, they briefly flashed a discrimination target at one of four possible locations. The discrimination performances were compared before and after the saccadic adaptation. Prior to adaptation, discrimination performance was best at the goal of the saccade. After adaptation, optimal discrimination performance was still observed at the goal of the first saccade, and not at the endpoint of the adapted saccade. This result is not consistent with OMRH/PMT, and Ditterich et al. concluded that the attentional focus is always directed to the primary target position and not to the saccade landing position [45]. However, Collins and colleagues argued that this conclusion was premature, given that the magnitude of the adaptation effects observed by Ditterich. was somewhat small. In two subsequent studies using more effective adaptation protocols they showed that saccadic adaptation does indeed produce adaptations of pre-saccadic attention [46,47] and that pre-saccadic displacement of attention would be shifted both to the position of the saccadic target and to the landing position of the adapted saccades [48]. In a recent study, Habchi and colleagues claimed that saccadic adaptation leads to changes in the allocation of covert attention, although these changes appear to be due to a more general bias towards the side of adaptation, rather than a modulation of covert orientation per se [49]. Overall, the evidence is consistent with the claim that saccadic adaption is associated with adaptations of pre-saccadic attention, which has been interpreted as evidence for OMRH/PMT.

Further evidence for OMRH/PMT is the finding that covertly attending a location produces a change in the trajectory of saccades, such that they deviate from the intended location [22]. Trajectories of vertical and oblique saccades are never completely straight but curvilinear, even when aiming at an isolated target [50,51], and it has been suggested that saccade curvature is determined by mechanisms situated in the final pathway of eye movement generation [52]. In addition to this natural tendency, other objects presented in the visual scene can influence the magnitude and direction of saccade curvature. Several authors have found that presenting an irrelevant distractor stimulus near a saccade target affects the saccade curvature [22,53,54,55]. In some instances, saccades can curve towards the irrelevant stimulus, as in visual search tasks [56], when the location of the saccade target is highly unpredictable, or for short-latency saccade [57], but in other cases, there is a tendency to deviate from the position of the distractor, particularly when saccade latencies are long [55], whether the saccade is reflexive or voluntarily triggered [53]. These trajectory deviations are typically attributed to competition between saccade plans associated with the target and distractor, and evidence that covert attention can also cause trajectory deviations [22,58,59,60] is therefore often cited as evidence for OMRH/PMT.

To briefly summarize, OMRH/PMT argues that covert orienting of attention depends on the activation of a saccade plan. Consistent with this hypothesis, there is a mandatory orientation of attention to saccade goals; covert and overt attention activate overlapping brain areas and damage to these areas causes problems with both overt and covert orienting. For example, ophthalmoplegic patients have deficits of covert attention that seem to mirror their ocular deficit. Moreover, modulating the gain of saccades also modulates the gain of pre-saccadic shifts of attention, and covertly attending a peripheral location affects the metrics of overt saccades, such that their trajectories are deviated away from the attended location. Altogether, these studies seem to offer clear evidence for a tight coupling between attention and oculomotor control.

## 3. The Case against OMRH/PMT

On first inspection, the evidence for OMRH/PMT seems overwhelming (e.g., [61]). However, we believe there are a number of reasons to be cautious about accepting these lines of evidence as conclusive proof of the claim that saccade programming *is necessary and sufficient* for covert orienting of spatial attention in the absence of an overt eye movement. Firstly, there is evidence that ‘pre-saccadic’ attention (i.e., the covert shift of attention that precedes an overt eye movement) is qualitatively different to covert attention. Secondly, although neuroimaging and some neuropsychological studies demonstrate associations between attention and oculomotor control, other studies have shown clear evidence of dissociations between saccade programming and covert orienting. Thirdly, behavioural studies that explicitly test the hypothesis that covert, endogenous attentional orienting is caused by saccade programming largely fail to support this hypothesis. Finally, while the evidence of interactions between saccade programming and covert attention suggests a relationship between the two processes, the evidence is not consistent with the claim made by OMRH/PMT, which is that covert orienting of attention is caused by activation of a saccade plan. We expand on these critiques in the following sections.

### 3.1. Pre-Saccadic Attention Is Not Equivalent to Covert Attention

The intention to make an eye movement produces radical changes in the receptive fields of neurons throughout the visual system, such that they appear to respond to stimuli in their post-saccadic spatial location before the saccade has begun [62]. This neurophysiological mechanism may well underpin the perceptual benefits observed in the moments before a saccade that are typically attributed to covert attention [63]. Critically, however, Duhamel et al. [62] also noted that this ‘pre-saccadic remapping’ did not occur when attention was deployed *without* a saccade, so cannot be responsible for ‘pure’ covert orienting (i.e., when the eyes remain fixated). If it is accepted that pre-saccadic remapping underpins pre-saccadic attention, and that pre-saccadic remapping only occurs prior to a saccade, it must also be accepted that pre-saccadic attention and ‘pure’ covert orienting of attention, which occurs when no saccade is executed, are served by a qualitatively different mechanisms

The proposal that pre-saccadic perceptual enhancements are qualitatively different to covert attention is consistent with neuropsychological evidence of a dissociation between covert attention and pre-saccadic perceptual enhancement. For example, Ladavas [64] asked patients with visual neglect to fixate and report target appearance using a button press response. Targets presented in the neglected field summoned involuntary eye movements on 45% of trials, but only half of these trials were associated with conscious detection of a target. When no saccade was made, only 4% of targets were detected. They concluded that the target could activate the oculomotor system without a concurrent shift of attention. In this case, the amplitude of the eye movements is not reported, so it is not clear whether the saccades that were not associated with target detection actually fixated the target (i.e., they might have fallen short, in which case the shift of attention could also have been hypometric). However, similar results were observed by Benson et al. [65] in a single case study of a patient with hemispatial neglect. In this study, a peripheral cue in the neglected hemifield summoned an eye movement but was not consciously detectible, again suggesting that the programming of eye movements and the orienting of attention can be dissociated. Blangero and colleagues [66] provided evidence of a double dissociation between the two processes. They reported the case of patient O.K., who presented with optic ataxia following a right parietal stroke, but no symptoms of neglect. Patient O.K. could make accurate saccades into the left hemifield and showed the typical pattern of pre-saccadic attentional enhancement at the saccade goal. However, the patient could not covertly attend to the same location when saccades were suppressed, demonstrating a dissociation between pre-saccadic attention and covert attention. Together, these studies suggest that pre-saccadic perceptual enhancements and covert orienting of attention are mediated by different cognitive mechanisms. If this proposal is correct, studies of pre-saccadic perceptual enhancement cannot be taken as evidence that shifts of covert spatial attention that occur in the absence of any overt eye movement rely on saccade programming.

### 3.2. Association Is Not Causation

The second main line of evidence in favour of OMRH/PMT draws on neurophysiological studies of non-human primates. These studies clearly showed that attention and eye movements share some common neural substrate and elegant work, showing that microstimulation of FEF leads to covert visual selection [37], is often presented as evidence for PMT. However, areas like FEF contain several distinct populations of neurons, some of which are involved in visual selection but not motor control, and others that are involved in saccade control but not visual attention [67,68,69]. Given that microstimulation of FEF may affect both visual and motor neurons [70], it is impossible to unambiguously attribute the attentional effects of microstimulation to the activation of motor programs. Furthermore, other research has shown that attending a stimulus does not affect the trajectory of microstimulation-evoked saccades [71], and concluded that covert attention is not necessarily associated with activation of a saccade plans, contrary to some of the behavioural findings reported in humans (e.g., [22]). A neurophysiological dissociation between saccade programming and covert orienting has also been observed using EEG in human participants by Weaver and colleagues [72]. The key finding here was that participants could endogenously allocate attention to the target object even on trials where the eyes were involuntarily directed to a salient distractor. This result is hard to reconcile with the claim that saccade preparation is both necessary and sufficient for covert attention. Overall, at best neurophysiological studies demonstrate an association between the brain areas required for saccade programming and those required for covert attention, and the few studies that offer a strong test of the key claim of PMT, which is that endogenous covert orienting is *caused* by saccade programming, seem to argue against this position (e.g., [71,73]).

### 3.3. Saccade Programming Does Not Necessarily Produce a Shift of Attention

OMRT/PMT argues that saccade programming produces shifts of attention. However, dual task experiments have repeatedly failed to observe attentional benefits at the goal of planned but unexecuted eye movements. In a seminal study by Klein [5] participants were asked to perform a variant of a go‒no-go task. In the majority of trials participants were instructed to prepare a saccade to the left or to the right, and execute the prepared saccade when an asterisk was presented at either the left or right location. Participants were faster at executing saccades when the peripheral onset was congruent with the saccade they had prepared. However, in occasional trials they were asked to cancel the saccade and make a manual response instead. The key finding here was that manual detection responses were not faster when probes appeared on the same side as they were instructed to prepare a saccade, suggesting that saccade programming led to shorter saccadic latencies but not a shift of attention. This result is incompatible with the claim that saccade programming is sufficient for covert orienting. A similar result was reported by Remington [74], who found that luminance detection was no better at a saccade goal than at a control location (although saccades were delayed when the luminance change occurred at the control location). Converging evidence for independence was provided by Stelmach and colleagues [75], using a Temporal Order Judgement (TOJ) task whereby two stimuli are sequentially presented with various inter-stimulus intervals, and participants are asked to indicate which stimulus appeared first. In this study endogenous attention to a peripheral location created a prior entry effect, such that the attended stimulus was perceived as appearing before the unattended stimulus. However, consistent with the findings of [5], planning a saccade to a peripheral location did *not* produce prior-entry, suggesting that this programming was not sufficient to orient attention. More recently, Born [76] used a stop-signal paradigm to confirm Klein’s claim that a saccade that is programmed but successfully inhibited does not produce a shift of attention.

Other studies have shown that saccades directed towards an intermediate position between two spatially close visual objects presented simultaneously in the periphery, referred to as ‘Global Effect’ [72,77,78], are not preceded by a shift of attention to the midpoint between stimuli. Rather, there is a subtle attentional enhancement at the location of both objects [73,79,80], even though the eventual eye movement lands at neither location. These observations appear to rule out the mandatory coupling of attention to the saccade landing point (but see Van der Stigchel and de Vries [81] for an alternative interpretation). Thus, the activation of a saccade program alone does not appear sufficient to elicit ‘covert’ orienting. In a related study, Bedard and Song used a visuomotor adaptation paradigm to dissociate the intended and actual endpoint of ballistic reaching movements [82]. They report that, in the post-adaptation phase, attention was allocated to locations associated with both the intended and the actual endpoint of movements, suggesting that endogenous covert attention can be decoupled from motor programs. In fact, there seems to be very little empirical evidence from human observers that preparing an eye movement is sufficient to produce a shift of attention when no saccade is executed.

Klein [5] conducted a second study to test the idea that attending a location was necessarily associated with the activation of a saccade program targeting the attended location. In this variant of the task, the primary response was a shift of attention, with saccades required on 20% of trials. The data show that attending a peripheral location produced faster manual responses but did not reduce saccade latency. Klein therefore concluded that covert orienting of attention and saccade programming were independent of one another. This conclusion was challenged by several authors, who argue that methodological factors, such as the requirement to make two speeded responses to peripheral events, mean the data are hard to interpret (e.g., [6]), but subsequent studies [8,83] addressed these issues and again found no evidence of attentional facilitation at the saccade goal. However, in a footnote Klein and Pontefract [8] noted that there was a long delay between the onsets of the cue and target, so it remained possible that saccade programming did elicit a shift of attention, just not at the time point measured by [5] or [8]. They speculate that OMRH/PMT might still be tenable for shifting, but not sustaining attention.

The idea that saccade programming could be sufficient for orienting but not for maintenance of attention was explicitly tested by Belopolsky and Theeuwes [84]. They observed that oculomotor priming effects were significantly reduced when a saccadic target is unlikely to appear at a cued location. Furthermore [9,84] demonstrated that participants could sustain attention at a location while simultaneously suppressing saccade programming to that same location. In these experiments, both exogenous and endogenous covert orienting were associated with the activation of a saccade motor plan. However, in the case of endogenous attention, the saccade execution was rapidly suppressed without disrupting the allocation of attention. In a recent study, we also observed that saccadic priming was profoundly affected by the probability that a saccadic response would be required by manipulating the proportion of catch trials in a cueing task. When there were many catch trials (30%), we observed covert orienting without saccadic priming, but when catch trials were removed there was both covert orienting and oculomotor priming [85]. Belopolsky and Theeuwes [84] proposed a revision to OMRH/PMT that they called a ‘Shifting and Maintenance (S&M) account of attention’. This revised theory, like that of Klein and Pontefract, retains the core assumption of OMRH/PMT that endogenous orienting depends upon a saccade motor plan but argues that once attention has moved an active saccade plan is not required to sustain attention. However, it is important to note that demonstrating an association between orienting of attention and the activation of a saccade plan is very different to demonstrating that the saccade programming causes orienting of attention. Indeed, this evidence is equally consistent with the idea that attentional selection is a necessary precondition for the programming of accurate saccades, as proposed by [14].

### 3.4. Impaired Oculomotor Control Disrupts Exogenous but Not Endogenous Covert Attention

Proponents of OMRH/PMT have studied patients with oculomotor problems and used ingenious experimental designs to experimentally constrain saccade programming. For example, Craighero et al. [41] argued that paralysis of the eye due to VIth nerve palsy was associated with deficits of covert, endogenous orienting. However, subsequent studies with both ophthalmoplegic patients and the eye-abduction paradigm reported results in conflict with Craighero and colleagues’ [41,42] observations. Smith, Rorden and Jackson [86] reported the case of A.I., who suffered from chronic ophthalmoplegia, a paralysis of the extraocular muscles that made her unable to make any eye movements. They observed a deficit of covert, exogenous attention with intact overt, endogenous orienting. Gabay and colleagues have shown similar effects in patients with Duane’s syndrome, a developmental disorder associated with problems making abductive eye movements [87]. The claim that defective oculomotor control is associated with impaired exogenous attention but preserved endogenous attention is consistent with observations in patients suffering from Progressive Supranuclear Palsy (PSP), a disease characterised by vertical paralysis of gaze [88]. For example, Posner et al. [89] examined covert orienting in PSP using a predictive, peripheral cue. When the stimuli were aligned along the horizontal axis, normal exogenous orienting was observed with a cue-target onset asynchrony (CTOA) of 50 ms. However, when the stimuli were aligned along the vertical axis covert orienting was not observed until a CTOA of 1000 ms, indicative of disrupted exogenous attention. Their subsequent study [90] explicitly compared exogenous and endogenous attention using non-predictive peripheral cues to engage exogenous attention and a centrally presented, predictive arrow cue to engage endogenous attention. As in the original study, there was a significant impairment of covert exogenous orienting when stimuli appeared along the vertical axis compared to the horizontal axis, whereas endogenous orienting was largely preserved along both axes. Furthermore, in a recent study we demonstrated that this selective impairment of exogenous orienting in PSP can also be observed in a visual search. More specifically, patients with PSP also suffer visual search deficits when targets appear on the vertical axis, and this deficit was greater for a feature search than a conjunction search [91].

The same dissociation between saccade planning and endogenous covert attention was observed in heathy participants using the eye-abduction paradigm [92]. We subsequently demonstrated that the effect of eye-abduction generalised to visual search, such that feature search was disrupted in the temporal hemispace while conjunction search was preserved [93,94]. Notably, the disruption of saccade programming associated with eye-abduction [95,96] and PSP [91] is also associated with a deficit of short-term spatial memory, which can be at least partly attributed to the failure to attend and encode the relevant locations. On the basis of these results, we concluded that the balance of evidence is more consistent with a weak view of OMRH/PMT that was only true for exogenous orienting.

An important caveat to this conclusion is that the interpretation of eye-abduction data is not entirely straightforward. Firstly, one might argue that participants can still plan eye movements even if they cannot be executed. However, an elegant experiment using eye-abduction demonstrated that the general tendency of saccades to curve away from a distractor location [53] was greatly reduced when the distractor was presented outside the oculomotor range [97]. Given that saccade curvature in a target-distractor paradigm is generally accepted to reflect competition between different saccade plans, this result strongly suggests that eye-abduction leads to impaired saccade programming. Secondly, and more problematically, the pattern of results is rather inconsistent. For example, in a follow-up study to Smith et al. [92], we examined the effect of eye-abduction on social attention (the reflexive shifts of attention triggered by observing an agent change their direction of gaze, also called ‘gaze-cueing’), non-predictive arrow cueing and peripheral cueing [98]. As in our previous study, we observed that eye-abduction interfered with covert exogenous orienting. However, in this study the interference effect was observed in the nasal, not the temporal hemifield. Furthermore, we also observed a reduced cueing effect in the nasal hemifield in the arrow cueing task. Interestingly, eye-abduction had no effect on gaze cueing, which was surprising given that gaze cues are known to activate the eye movement system [99,100]. In addition, although not directly relevant to OMRH/PMT, Michalczyk et al. [101] recently observed that eye-abduction disrupted IOR, a result contrary to our 2012 finding. The precise reasons for these disparate findings are not entirely clear. We attributed the nasal-hemifield effect to a reduction in the cost of invalid cues, but as MacLean et al., observed, multiple interpretations are possible, which limits the strength of the conclusions we can draw based on eye-abduction [102]. Given that studies using eye-abduction only use a single Stimulus Onset Asynchrony (SOA), it is also possible that exogenous orienting was delayed by eye-abduction rather than completely abolished in these tasks (as was the case in the studies of patients with PSP [89,90]). A final problem is that eye-abduction creates a very unusual oculoproprioceptive signal, and there is some evidence that oculoproprioception plays an important role in spatial attention (e.g., [103]). It is therefore possible that the impaired attentional orienting observed in ophthalmoplegic patients and studies of eye-abduction was caused by disrupted oculoproprioception, rather than impaired saccade programming per se.

**Figure 3:**
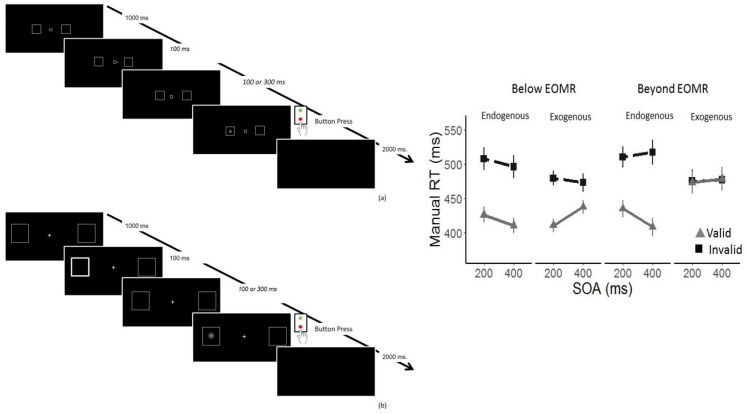
The sequence of events in the endogenous cueing (**a**) and exogenous cueing (**b**) tasks. The right panel shows the mean manual reaction time (RT) in ms as a function of Stimulus Onset Asynchrony (SOA) and cue validity for below and beyond the EOMR separately for the endogenous and exogenous cueing task (Exp. 3). Adapted from [103].

In order to address these issues and provide a more rigorous test of exogenous-only version of OMRH/PMT we developed a new variant of the Posner cueing task in which cues and targets were presented within or beyond the effective oculomotor range (EOMR) [104]. Eye-abduction is thought to induce biased proprioception [105], which could lead to a bias in attention, although a recent study has cast some doubt on this claim [106]; we nevertheless used Presentation in Extreme Periphery (PEP), which has the advantage of presenting stimuli in the far periphery (up to 44° of visual angle) while keeping the participant’s eye and trunk in their canonical, natural position. Each participant’s Effective Oculomotor Range (EOMR) was computed in order to define the location of the placeholders in the different cueing tasks. In all three experiments reported, the target and placeholders could appear either below or beyond the participants’ EOMR. The first experiment examined exogenous, covert orienting using a peripheral cueing task and SOAs of 100, 200, 300 or 500 ms. Consistent with studies with patients [86,87] or with the eye-abduction paradigm [90,92,94], exogenous cueing effects were abolished at all SOAs when stimuli were presented beyond the participant’s EOMR, but intact when stimuli appeared within the EOMR. In a second experiment, we tested endogenous covert attention using a predictive, central cue. As with previously reported experiments [86,87,90,92,94], but, contrary to [41,42], there was no deficit in attention when stimuli were presented beyond EOMR. In a third experiment, we tested both exogenous and endogenous attention using a within-participant design. In accordance with the first two experiments, exogenous, covert orienting to peripheral cues was disrupted when targets appeared beyond the EOMR, whereas covert endogenous orienting was preserved (see Figure 3). In a recent experiment we replicated this dissociation using visual search tasks, such that a ‘pre-attentive’ search for feature singletons (which relies on the same cognitive processes as exogenous attention) was only possible within the effective oculomotor range. When feature search arrays were presented beyond the EOMR, participants had to engage in serial, attentive searching to find the target [107]. These findings rule out the possibility that previous reports of dissociations between endogenous orienting and saccade programming can be explained in terms of abnormal oculoproprioception or in terms of delayed, rather than abolished, covert orienting.

### 3.5. Saccades Curve away from Attended Locations

The observation that saccade trajectories are affected by covert attention is typically interpreted as evidence in favour of OMRH/PMT (e.g., [59]). However, this interpretation is problematic, because the studies classically report that saccades tended to curve *away* from the cued location [58]. The standard interpretation for this effect is that participants need to inhibit the programmed eye movement to the cued location in order to be able to execute a saccade towards the target location [53,108]. Saccade curvature will depend on the distribution of neuronal activation produced both cue and target. A curvature *away* from the cued location would result from an inhibition of the neurons coding for the irrelevant cued position, allowing the neuronal population coding for the actual target location to take over. This inhibition is thought to come more particularly from projections from the Frontal Eye Field (FEF) and Superior Colliculus (SC) (see [109]). This explanation is consistent with the broad idea that covert attention and motor programming interact. However, it is much harder to reconcile with the specific claim made by OMRH/PMT that shifts of attention are *caused* by motor programs. In fact, the observation that covert, endogenous attention can be allocated to a location that is currently inhibited in the oculomotor system is the *opposite* of what is predicted by OMRH/PMT. Studies of saccade trajectory deviations therefore demonstrate an interaction between covert attention and saccade preparation, but do not provide convincing evidence that covert orienting of attention is caused by the activation of a saccade plan and therefore do not support OMRH/PMT. Furthermore, although the mechanisms underlying curvature towards a distractor are clearly understood [109,110], there is less consensus regarding mechanisms underlying curvature away from distractor. For example, curvatures away from and irrelevant position are observed when participants have a prior knowledge of target position [55], and the direction of the deviation appear to be dependent on response time, such that short latency saccades tend to deviate towards an irrelevant position, whereas slow saccades tend to deviate away [111]. This observation suggests that the oculomotor inhibition operates in the selection process, leaving plenty of time for top-down preparation. Hence, the deviation away observed in the case of covert endogenous shift of attention cannot be explained solely in terms of activation of the oculomotor system.

## 4. An Oculomotor Readiness Hypothesis of Exogenous Orienting (OREO)

On the basis of these studies, we argue that the data are most consistent with an Oculomotor Readiness Hypothesis that is specific to Exogenous Orienting (OREO). On a theoretical level, the relationship between attention and eye movements can be understood in terms of Biased Competition, such that activation of the motor system exerts a powerful biasing influence on competitive interactions in the visual system [112]. In Biased Competition, the locus of attention arises from a stimulus-driven competition between signals relating to stimulus salience (e.g., their brightness, size, contrast, orientation), which can be biased by goal-driven factors such as the goals of the observer. The competition takes places in a topographic map of space, called a priority map ([113]. The cortical substrates of the priority map are thought to lie in the posterior parietal cortex a region that has dense reciprocal connections with areas known to be directly involved in saccade control such as Frontal Eye Field (FEF) and Superior Colliculus (SC) (for a review, see [114]) When a location is activated in the priority map the activation is passed downstream to oculomotor structures, such as the SC, which represent the prioritized location as the goal of a potential movement. These oculomotor signals are then fed back into the priority map, thus further biasing activity in favour of the activated location [115]. This reciprocal feedback loop will typically produce very rapid selection of a peripherally cued location, which will facilitate target detection, producing the rapidly developing perceptual advantage typically associated with exogenous attention. When the oculomotor system malfunctions, or when targets appear at locations that cannot become the goal of a saccade, the motor system exerts a much weaker influence on the biased competition. If a target is associated with a persistently large salience signal (e.g., in a feature search task in which the search array remains visible until a response is made), the absence of reciprocal reinforcement from the oculomotor system should slow selection of the feature singleton but will not necessarily prevent its selection. This is exactly the pattern we observed, such that placing a salient feature beyond the EOMR delayed, rather than abolished the capture of attention by the singleton [93,94,103]. If salience signal is transient (as in the peripheral cueing task), the absence of reinforcement from the oculomotor system reduces the chance of the cued location ‘winning’ the competition before the signal decays, and therefore reduces the probability of observing an exogenous shift of attention to the cued location. We can therefore understand the relationship between exogenous attention and saccade programming in terms of oculomotor inputs that bias competition on the priority map in favour of the saccade endpoint. The demotion of the oculomotor system from being the sole arbiter of the locus of attention to being one of many potential influences on the process of biased competition is a key difference between OREO and OMRH/PMT. Importantly it does not deny the possibility that exogenous orienting can be driven by other inputs, such as stimulus salience [116]. Rather, OREO holds that optimally efficient exogenous orienting relies on activation of a saccade plan, and when this activation is disrupted exogenous orienting becomes slower and less reliable.

OREO makes some clear and testable predictions about the interaction between covert, exogenous orienting and saccade programming. Firstly, exogenous orienting should always be associated with the activation of a saccade plan. Secondly, inability to plan a saccade should disrupt exogenous orienting. Thirdly, factors that affect the properties of saccadic eye movements (e.g., their latency, amplitude and direction) should also affect the speed and accuracy of covert exogenous orienting. MacLean et al. [102] tested the first prediction using a variant of the dual task procedure developed by Klein and Pontefract [8]. Contrary to the predictions of OREO, they observed no reduction in saccade latency at peripherally cued locations and concluded that exogenous orienting was not associated with saccade programming. However, this conclusion is premature, as MacLean et al. used a SOA of 250 ms, allowing ample time for the suppression of saccade programming following a shift of attention. Indeed, the authors concede that their results are more similar to those of Belopolsky and Theeuwes [9,84], who previously argued that maintenance of attention was independent of saccade programming. The MacLean study also utilises a very high proportion of ‘no-go’ trials, where a cue appears but no saccade is permitted, and as we have already noted, a high proportion no-go trials can mask saccadic priming effects caused by peripheral cues [9,107]. We examined the third prediction by using instrumental conditioning of eye movements [117]. If exogenous orienting depends on activation of the oculomotor system, then one might predict that a manipulation that modulates saccade latencies should also affect covert exogenous attention. In our first experiment we found that rewarding eye movements to a specific spatial location reliably reduced saccade latencies to that location, and that this conditioning persisted for 180 trials once rewards were removed. However, in a second experiment this modulation of the oculomotor system had no effect on the magnitude of covert, exogenous orienting or Inhibition of Return. McCoy and Theeuwes [118] report a similar result in a study in which participants learned to made saccades to a location associated with a large reward. As with our study, the high-value location was associated with shorter saccade latencies. However, this oculomotor facilitation did not translate into enhanced performance at the rewarded location in a subsequent task that measured perceptual discrimination at the rewarded location while the eyes remained at fixation. These findings may seem hard to reconcile with the third prediction of OREO, but it is important to note that OREO predicts that reducing the latency of a saccade should lead to a reduction in the rise-time of attention (i.e., the speed at which attention is oriented to the cued location) rather than the absolute magnitude of the cueing effect. Thus, in our view, none of these studies offers a strong test of the predictions of OREO. In contrast, McFadden, Khan and Wallman [119] reported that it was possible to elicit adaptation of exogenous, covert orienting, which was accompanied by an adaptation of subsequent eye movements, suggesting that the adaptation of exogenous attention relied on changes in the oculomotor plans elicited by the peripheral onset. It is not known whether endogenous, covert attention can be adapted in the same way, but such a study would provide a good test of OMRH/PMT and OREO, and the former theories predict an effect of adaptation of endogenous attention on saccade amplitude, whereas OREO does not.

## 5. Summary and Conclusions

To briefly summarize, OMRH/PMT argues that planning an eye movement is both necessary and sufficient for covert, endogenous orienting of attention. Many studies suggest that there is an association between covert attention and oculomotor control, but none of this evidence demonstrates a causal relationship between saccade programming and covert, endogenous spatial attention. Studies of pre-saccadic attention are problematic because they conflate peri-saccadic perceptual changes (‘remapping’) with covert attention, and the results are equally consistent with the view that orienting attention is a necessary precondition for saccade programming (e.g., [120]). A single neuropsychological study argues for an association between endogenous orienting and saccade programming, but there are many other examples of double dissociations between oculomotor control and endogenous, covert attention. Studies of healthy participants show no evidence that shifts of attention can be achieved by programming an eye movement, and the weight of evidence from eye-abduction and other manipulations suggests that endogenous covert orienting can be achieved in the absence of saccade programming. Overall, there is surprisingly little evidence from human participants that saccade programming is either necessary or sufficient for covert spatial attention. However, there is a growing body of neuropsychological and experimental evidence that exogenous covert orienting is dependent on the ability to plan and execute eye movements. Neuropsychological patients with paralysis of the eyes reliably present with deficits of exogenous, covert attention and disrupting saccade programming in healthy participants interferes with covert, exogenous orienting. In our view, these findings are powerful and conclusive evidence against the central tenet of OMRH/PMT, which is that saccade programming is necessary and sufficient for endogenous, covert orienting, and thus we should reject OMRH/PMT as a theory of covert, endogenous attention. However, the data are consistent with OREO, which holds that saccade preparation or ‘oculomotor readiness’ plays a fundamental role in covert, exogenous orienting of attention.
